# Involvement of the Chemokine Prokineticin-2 (PROK2) in Alzheimer’s Disease: From Animal Models to the Human Pathology

**DOI:** 10.3390/cells8111430

**Published:** 2019-11-13

**Authors:** Roberta Lattanzi, Daniela Maftei, Carla Petrella, Massimo Pieri, Giulia Sancesario, Tommaso Schirinzi, Sergio Bernardini, Christian Barbato, Massimo Ralli, Antonio Greco, Roberta Possenti, Giuseppe Sancesario, Cinzia Severini

**Affiliations:** 1 Department of Physiology and Pharmacology “Vittorio Erspamer”, Sapienza University of Rome, P.za A. Moro 5, 00185 Rome, Italy; 2 Institute of Biochemistry and Cell Biology, IBBC, CNR, Viale del Policlinico, 155, 00161 Rome, Italy; 3 Department of Experimental Medicine and Surgery, University of Rome Tor Vergata, 00133 Rome, Italy; 4 Neuroimmunology Unit, IRCCS Santa Lucia Foundation, v. Ardeatina 354, 00179 Rome, Italy; 5 Department of Systems Medicine, University of Rome Tor Vergata, 00133 Rome, Italysancesario@med.uniroma2.it (G.S.); 6 Department of Sense Organs, University Sapienza of Rome, Viale del Policlinico 155, 00161 Rome, Italy

**Keywords:** Alzheimer’s disease, neuroinflammation, chemokines, prokineticins, blood biomarkers

## Abstract

Among mediators of inflammation, chemokines play a pivotal role in the neuroinflammatory process related to Alzheimer’s disease (AD). The chemokine Bv8/prokineticin 2 (PROK2) is a critical player in inflammatory and neuroinflammatory diseases and has been demonstrated to be involved in Aβ toxicity. The aim of the present study was to extend the research to rats chronically intracerebroventricularly (i.c.v.) injected with Aβ, to an AD transgenic mouse model, and subsequently to AD patients, mainly with the aim of detecting a potential biomarker. Real-time PCR and immunofluorescence analysis were used to evaluate Prokineticin-2 (PROK2) mRNA and the corresponding protein levels in both animal and human AD brain extracts, and the ELISA test was used to measure the amount of PROK2 in the serum of AD patients. We demonstrated a significant upregulation of PROK2 levels in brain tissues of Aβ_1–42_ i.c.v. injected rats, transgenic AD mice (Tg2576), and in the hippocampus of AD patients. Additionally, through a pilot study, an approximate twofold increase of PROK2 levels has been proved in the serum of AD patients, compared to the control subjects, identifying a potential blood-based biomarker of the disease.

## 1. Introduction

Alzheimer’s disease (AD) is the widespread neurodegenerative disease, mainly characterized by the extracellular deposition of misfolded amyloid beta (Aβ) proteins in amyloid plaques and by intraneuronal tangles composed by the hyperphosphorylated tau protein [1].

Over the past few decades, a role played by the immune system in AD has been highlighted, as demonstrated by the presence of activated microglia and astrocytes in the brains of affected patients [2,3]. Indeed, deposition of aberrant proteins is responsible for an immune reaction against proteins that trigger a neuroinflammatory response [4]. Neuroinflammation in AD is present at both a parenchymal level and at the barriers of the brain, increasing with the ongoing deposition of Aβ and the disease progression [3].

However, correlative analyses of the clinical symptoms that precede AD, namely mild cognitive impairment (MCI), and the presence of inflammatory mediators in the cerebrospinal fluid (CSF) have indicated a much earlier involvement of the immune system [5,6].

Among mediators of inflammation, chemokines play a pivotal role in the neuroinflammatory process due to their dual function as both chemoattractants for immune cells and molecular messengers in crosstalk among central nervous system (CNS)-resident cells. In fact, it has been demonstrated that chemokines play a critical role in the onset and progression of neuroinflammation, by affecting the functions of various cell populations, including resident glial cells, and by infiltrating immune cells [7,8,9].

Recent data suggest that among pro-inflammatory signal molecules released by astrocytes and microglia in AD pathology, the chemokine system (chemokine ligands and their cognate receptors) participates in the immune response in AD brain [8,10].

Chemokines are small heparin-binding proteins (8–14 kDa), primarily known for their role in the attraction of circulating leukocytes to sites of inflammation or injury during immune responses [11]. There are data suggesting that some chemokines may represent promising biomarkers of AD, since their levels appeared significantly modified in the blood or CSF of AD patients.

Included in the chemokines family, elevated CX3CL1 (fractalkine) plasma levels have been found in mild cognitive impairment (MCI) and mild AD patients [12], as well as an increase of *CX3CL1* gene expression in the brains of AD patients in respect to control subjects, mainly in the early stage [13].

Moreover, changes in the expression of chemokine receptors on peripheral blood cells of AD patients have also been reported, suggesting an influence of the pro-inflammatory milieu in AD [14,15].

Furthermore, a very recent international multicentric study, analyzing 53 inflammatory proteins in plasma of both AD and MCI, identified eotaxin-1 (higher in AD plasma vs. CTRL and MCI) and Monocyte Chemoattractant Protein-1 (MCP-1 or CCL2) (lower in AD plasma vs. CTRL and MCI), as potential inflammatory AD biomarkers [16].

These data support the importance of such small proteins, mediators of neuroinflammation, as a tool for supporting AD diagnosis and to better differentiate patients from healthy controls.

A family of chemokines, Bv8/prokineticin (PROK2), interacting with two G-protein coupled receptors (PKR_1_ and PKR_2_), has recently emerged as a critical player in immune system and inflammatory diseases [17,18].

It has been demonstrated that PROK2 expression is upregulated by several pathological stressors, including hypoxia and reactive oxygen species. It has also been shown that PROK2 is an insult-inducible endangering mediator for cerebral ischemic injury, supporting a role of this chemokine in brain pathological states [19,20].

More recently, our team established that Aβ, both in vitro, in neuron and astrocyte primary cultures, and in vivo, in intracerebroventricularly (i.c.v.) injected rats, was able to upregulate the prokineticin system early on, indicating a well-defined involvement of PROK2 in Aβ toxicity. Moreover, we demonstrated that the pharmacological blockade of the prokineticin system can exert neuroprotective effects by preventing PROK2 upregulation, thus suggesting that this system may represent, at least in animal models, a new pathological hallmark for AD [21,22,23].

Considering the involvement of PROK2 and its receptors in Aβ toxicity, the present study aimed to extend our research to rats chronically i.c.v. injected with Aβ, to the AD transgenic mouse model Tg2576, and subsequently to AD patients, mainly with the aim of detecting a potential biomarker.

## 2. Materials and Methods

### 2.1. Chemicals

Aβ_1–42_ (Abcam, Cambridge, UK) was dissolved in sterile phosphate buffer (PBS; 0.01 M NaH_2_PO_4_, 0.15 M NaCl, pH 7.4) at a concentration of 1 mg/mL and stored at −20 °C. Before in vivo injection, aliquots of Aβ peptide were aggregated by incubation at 37 °C for 72 h [24].

Human brain material was provided via the rapid autopsy program of the Netherlands Brain Bank (NBB), which provides post-mortem specimens from clinically well documented and neuropathologically confirmed cases.

### 2.2. Animals

Ethics statement: The experiments which involved animal care and treatment were performed in accordance with the guidelines of the European Communities Council (2010/63/UE) and the protocol was approved by the Animal Care and Use Committee of the Italian Ministry of Health (authorization number: 79/2015-PR).

Experiments were conducted on adult male Sprague–Dawley rats supplied by Charles River (Charles River, Como, Italy) weighting at the time of surgery 280–320 g, and on transgenic AD mouse line TG2576 and their non-transgenic littermates (Taconic Europe, Lille Skensved, Denmark). Animals were group housed under 12:12 h light/dark cycles, 21 ± 2 °C temperature and 50–60% humidity, with access to food and water ad libitum.

### 2.3. Surgery and Aβ_1–42_ Administration

Anesthetized rats (*n* = 30 rat) (ketamine–xylazine, 60 + 10 mg/kg intraperitoneally, i.p.) were placed in a stereotaxic surgery apparatus and implanted with a plastic guide cannula (Linca, Tel-Aviv, Israel), inserted into the left lateral ventricle through a hole drilled over the skull at the following coordinates: antero-posterior, −1 mm; lateral, +1.8 mm relative to bregma, according to the atlas of Paxinos and Watson [25]. The cannula was secured to the skull bone with dental cement. One week after the implantation, rats were randomly assigned to two groups and i.c.v. injected with Aβ_1–42_ (1 μg/μL; 5 µL injection volume) or its vehicle (PBS; 5 µL injection volume). A 10 μL Hamilton syringe fitted with a 26-gauge needle was used for the injection. The syringe was inserted through the guide cannula to a depth of 4.2 mm below the external surface of the skull and at the end of the injection the needle was left in place for 10 s to avoid reflux of the solute.

### 2.4. RNA Extraction and Real-Time PCR

Total RNA was isolated from homogenized prefrontal cortex and hippocampal samples using the Trizol reagent (Sigma Aldrich, St. Louis, MO, USA) following manufacturer’s instruction. The RNA concentration was determined by spectrophotometry and the sample’s purity was evaluated according to 260/280 nm ratio. One microgram of total mRNA was reverse transcripted (Reverse Transcriptase, Promega, Madison, WI, USA) to obtain cDNA, which was further used as a template in real-time PCR (iCycler; Bio-Rad, Hercules, CA, USA). All reactions were performed in triplicate using iQ SYBR Green Supermix (Bio-line, London, UK), under the following conditions: 95 °C for 10 min (polymerase activation) followed by 40 cycles at 95 °C for 30 s, 56–50 °C (temp. depends on the Tm of primers) for 30 s and 72 °C for 30 s. A reaction mixture without cDNA was used as control. The results were quantified using the comparative threshold method. The Ct value of the specific gene of interest was normalized to the Ct value of the endogenous control, glyceraldehydes-3-phosphate dehydrogenase (GAPDH) for mouse and rat samples, and β-actin for human samples. The comparative Ct method (2^-ΔΔCt^) was then applied using control as the reference samples. The primer sequences were reported in Table 1.

### 2.5. Immunofluorescence

Immunofluorescence staining was performed as previously described [23]. Brains were removed from intracardially perfused rats (PBS followed by 4% paraformaldehyde, PFA), post-fixed for 24 h in the same fixative at 4 °C and cryoprotected in 30% sucrose solution at 4 °C until they sank. Thereafter, brains were serially cut using a cryostat (Leica Microsystems Nussloch GmbH, Heidelberger, Germany) to obtain coronal sections (40 μm).

After the block of non-specific sites with 3% normal donkey serum for 1 h at room temperature (RT) the brain sections were incubated at 4 °C for 48 h with the following primary antibodies: rabbit polyclonal anti-PROK2 (1/200, Abcam, Cambridge, UK, ab76747), mouse monoclonal anti-neuronal nuclei (1/500, NeuN, MAB-90228, Immunological Sciences, Rome, Italy), mouse monoclonal anti-glial fibrillary acidic protein (1/400, GFAP, MAB-12029, Immunological Sciences, Rome, Italy). Thereafter, sections were washed and incubated for 2 h at RT with anti-species IgG secondary antibodies coupled to Alexa Fluor^®^-488 or -555 (1:200, Immunological Sciences). The immunostaining specificity of the secondary antibodies was detected by incubating control sections with the secondary antibodies alone. DAPI (4′,6-diamino-2-phenylindole, 1/500, Sigma Aldrich) was used to stain the nuclei. The immunofluorescence images were acquired with a confocal laser scanning microscope (Leica SP5, Leica Microsystems, Wetzlar, Germany) by an experimenter who was blind to the experimental design.

The acquisition settings were maintained identical among the different experimental cases. For figure production, brightness and contrast of images were adjusted by applying the same values, and by taking care to leave a tissue fluorescence background for visual appreciation of the whole fluorescence intensity features and to help comparison between the different experimental groups. Final figures were assembled using Adobe Photoshop 7 and Adobe Illustrator 10.

### 2.6. In Vivo Blood Assay

A total of 40 subjects, grouped in AD (*n* = 20) and controls (CTRL *n* = 20), were recruited at the Neurology Unit of Tor Vergata University Hospital, Rome, Italy, between 2016 and 2017.

AD was diagnosed according to 2011 criteria [26]. All of the AD patients underwent standard diagnostic work-up for dementia, including medical examination, laboratory tests, electroencephalogram (EEG), neuropsychological tests, brain Magnetic Resonance Imaging (MRI), Positron Emission Tomography with 2-deoxy-2-[fluorine-18]fluoro-d-glucose integrated with Computed Tomography, [^18^F] FDG PET, and lumbar puncture (LP) for CSF analysis. According to previous works, CSF biomarkers Aβ_1–42_, p-tau, and total tau were used to improve diagnostic accuracy [27]. Patients showing abnormal CSF cell count (>4 cells/µL), acute and/or chronic systemic conditions, other neurological diseases were excluded from the study. Control subjects were age/sex matched healthy individuals, enrolled among non-blood relatives of AD or volunteers, which did not have family/medical history and signs of neurological diseases.

Enrolled subjects underwent venous blood sampling. Specimens were collected in standardized conditions, in the morning, after overnight fasting. Procedure was conducted in a comfortable room, after the signature of informed consent. Immediately after collection, serum was aliquoted and stored at −80 °C.

### 2.7. ELISA

Serum PROK2 levels from AD patients (age 80 ± 2) and control subjects (age 76 ± 4) were assayed using a competitive commercial ELISA kit (Life Span BioSciences, Inc., Seattle, WA, USA) according to the manufacturer’s instructions. The ELISA test was performed on 96-well microplates by experimenters who were blind to the experimental design. Each sample was run in triplicate with <5% intra-assay and <9% interassay coefficients of variation, expressing results as pg/mL.

### 2.8. Data Analysis

Statistical analysis was performed using SPSS 11.0.0 for Windows (SPSS Inc., Chicago, IL, USA) or GraphPad Prism 6. All results are expressed as mean ± SEM, with *n* as the number of independent samples. The significance was performed by one-way analysis of variance (ANOVA) followed by Bonferroni’s test for multiple comparisons or by Student T test. The significance level was set at *p* < 0.05 (*), *p* < 0.01 (**), and *p* < 0.001 (***).

## 3. Results

### 3.1. Upregulation of PROK2 mRNA in Rat Brain Following Aβ Insult

**Figure 1:**
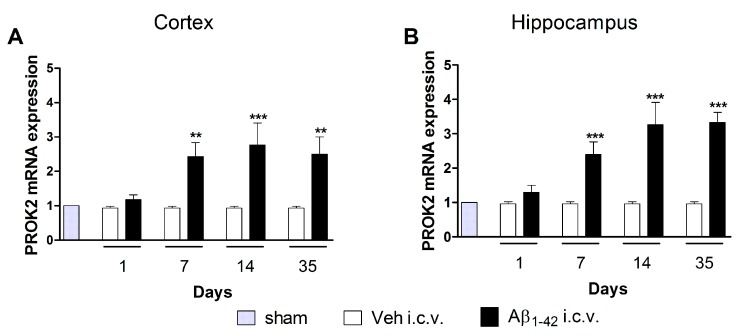
mRNA expression level of BV8/prokineticin (PROK2) in rat cortex and hippocampus. Relative PROK2 mRNA expression levels were determined by qRT-PCR in cortex (**A**) and hippocampus (**B**) of vehicle and Aβ_1–42_ injected rats at different time points: 1, 7, 14, 35 days after injection. The mRNA expression levels were expressed in relation to glyceralde-hydes-3-phosphate dehydrogenase (GAPDH) and presented as fold of increase respect to sham rats. Data represent means ± SEM of three separate experiments. Statistical analyses were performed using one-way ANOVA test followed by Bonferroni’s post hoc test. ** *p* < 0.01; *** *p* < 0.001; Aβ_1–42_ intracerebroventricularly (i.c.v.) vs. Veh i.c.v.

We have previously demonstrated that i.c.v. injection of Aβ_1–42_ induced a significant increase in PROK2 mRNA levels in rat cortex and hippocampus at early time points (6–48 h after injection) [21]. Here, we investigated the long-term modulation of PROK2 expression induced by Aβ_1–42_ injection analyzing mRNA levels of PROK2 after 1, 7, 14, and 35 days in extracts of cortex and hippocampus of treated rats. Time-course analysis of mRNA expression displayed a remarkable modulation of PROK2 in both cortex and hippocampus of Aβ_1–42_ injected rats. As shown in Figure 1, PROK2 mRNA levels were significantly increased starting from day 7 after i.c.v. injection and remained at the same level up to 35 days later, thus indicating a long-lasting effect of Aβ in the prokineticin system modulation.

### 3.2. Localization of PROK2 Protein in Rat Brain Following Aβ Insult

**Figure 2:**
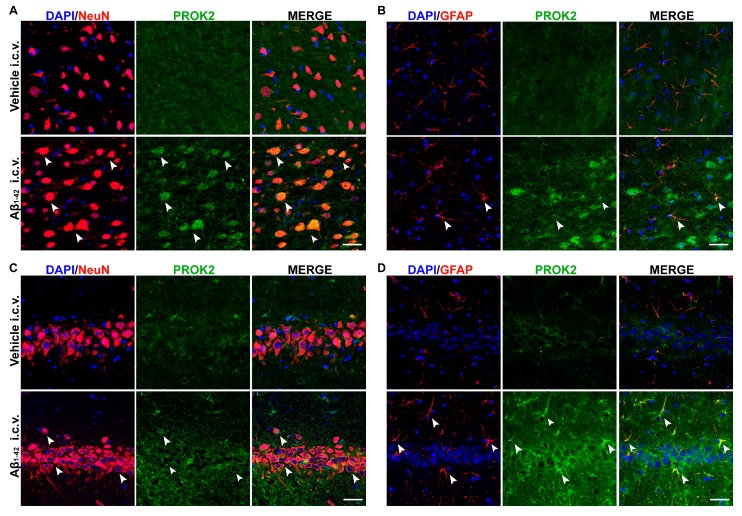
Immunofluorescence analysis of PROK2 expression in rat cortex and hippocampus. Representative confocal images of rat cortex (**A**,**B**) and hippocampus (**C**,**D**) stained with anti-PROK2 antibody (green) 35 days after Vehicle or Aβ_1–42_ i.c.v. injection. Neurons were stained with NeuN (red), astrocytes with glial fibrillary acidic protein (GFAP) (red) and cell nuclei were stained with DAPI (blue). PROK2 immunofluorescence signal was undetectable in the cortex (**A**,**B**, first row) and hippocampus (**C**,**D**, first row) of vehicle infused rats. Aβ_1–42_ i.c.v. injection strongly increased PROK2 immunofluorescence signal in neurons and astrocytes, in both cortex and hippocampus, as demonstrated by the colocalization with NeuN (arrow, **A**,**C**, second row) and GFAP (arrow, **B**,**D**, second row). Scale bar, 30 μm.

As previously established by immunofluorescence in primary mixed cortical cultures, Aβ_1–42_ treatment time-dependently induced upregulation of PROK2 both in body neurons (NeuN^+^ cells) and in astrocytes processes (GFAP^+^ cells) [21]. Here, we investigated the localization of PROK2 protein in cortex and hippocampus of rats 35 days after Aβ_1–42_ or vehicle injection. As shown in Figure 2, PROK2 immunofluorescence signal (green) appeared almost undetectable in the cortex and hippocampus of vehicle infused rats, while Aβ_1–42_ i.c.v. injection strongly increased PROK2 expression in neurons and astrocytes, as demonstrated by the co-localization with NeuN and GFAP, respectively. By contrast, we did not observe any increase in PROK2 immunoreactivity in microglia (data not shown).

### 3.3. Upregulation of PROK2 in Tg2576 Brain

**Figure 3:**
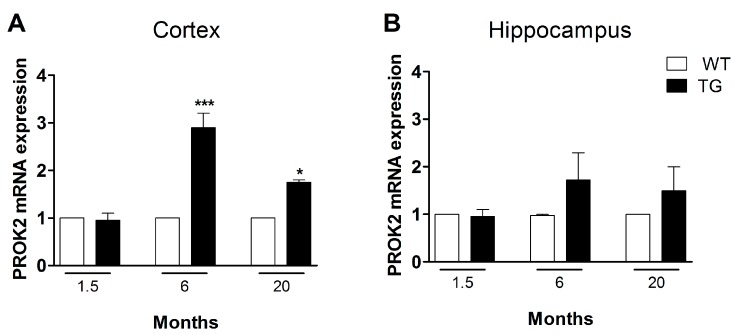
mRNA expression level of PROK2 in Tg2576 cortex and hippocampus. Relative PROK2 mRNA expression levels were determined by qRT-PCR in cortex (**A**) and hippocampus (**B**) of wild type (WT) and Tg2576 mice at the age of 1.5, 6, and 20 months. The mRNA expression levels were expressed in relation to GAPDH and presented as fold of increase respect to WT animals. Data represent means ± SEM of five animals. Statistical analyses were performed using one-way ANOVA test followed by Bonferroni’s post hoc test. * *p* < 0.05, *** *p* < 0.001 Tg2576 vs. WT.

We analyzed, by qRT-PCR, PROK2 mRNA levels in cortical and hippocampal tissues from transgenic AD animals (Tg2576 mice, TG) at different ages (1.5, 6, and 20 months), comparing them to wild-type (WT) control mice. As shown in Figure 3A, in cortical tissues of six-month-old animals (pre-symptomatic stage) and at 20 months a significant PROK2 upregulation was detected, thus confirming results obtained in the non-transgenic AD animal model (Aβ i.c.v. injected rats). Of note, the increase in PROK2 mRNA amount appeared higher in the pre-symptomatic stage, i.e., in six-month-old mice (2.9 ± 0.3 fold with respect to their non-transgenic CTRL) and lower in the more advanced stage of the pathology, i.e., in 20-month-old mice (1.75 ± 0.05 fold with respect to their non-transgenic CTRL). Moreover, in the hippocampus a PROK2 increase was observed, in both six- and 20-month-old mice, even if this increase was not statistically significant (Figure 3B).

### 3.4. Upregulation of PROK2 in AD Patients’ Brain

**Figure 4:**
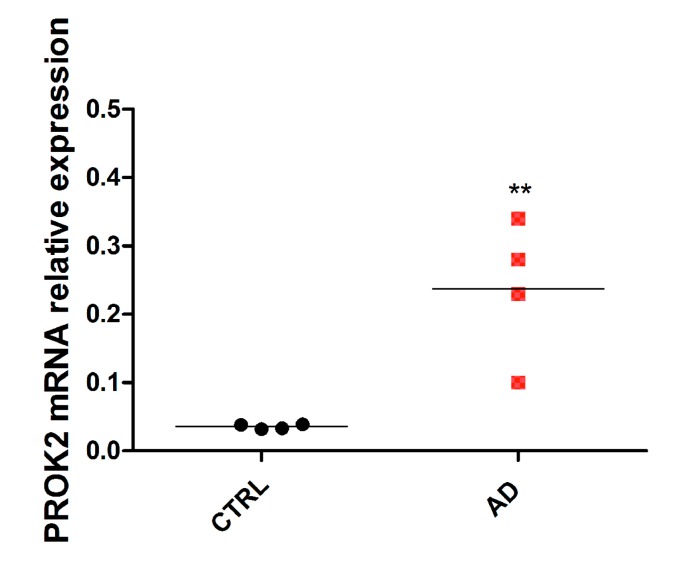
mRNA expression level of PROK2 in human hippocampus. Relative PROK2 mRNA expression in hippocampus from control subjects (without neurologic and/or neurodegenerative and/or inflammatory disease) and in patients with diagnosed Alzheimer’s disease (AD) (with overt clinical and cognitive decline) (*n* = 4; ** *p* < 0.01 by Student T test).

Subsequently, we have extended our observations to AD human brain by qRT-PCR analysis of post-mortem hippocampal tissues obtained by the Netherlands Brain Bank (NBB). As shown in Figure 4, a significant PROK2 mRNA upregulation (of about six times) was observed in hippocampal regions from AD patients, as compared to age-matched cognitively intact human controls.

### 3.5. Increase of PROK2 in AD Patients’ Serum

**Figure 5:**
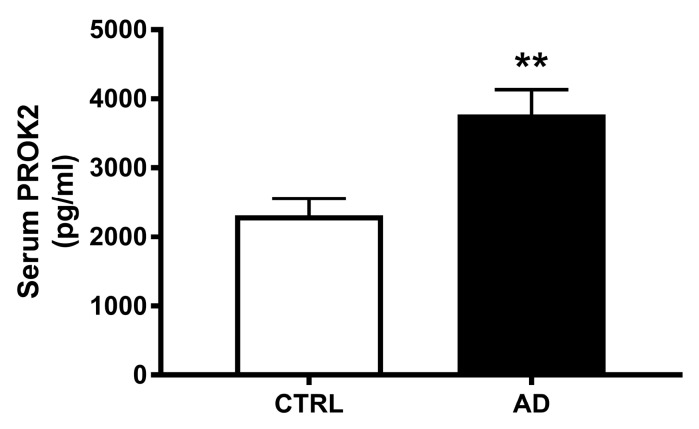
Serum levels of PROK2 in control subjects and AD patients. Levels of PROK2 obtained by ELISA test (*n* = 20; ** *p* < 0.01 by Student T test).

Analyzing PROK2 in the serum of 20 AD patients and 20 control subjects we found a significant PROK2 increase in patients. As shown in Figure 5, quantitative analysis revealed an about 1.6-fold increase of this chemokine level in serum samples obtained from AD patients, compared to samples from control subjects (*p* < 0.001).

## 4. Discussion

Chronic neuroinflammation in AD is accompanied by persistent activation of astrocytes and microglial cells and by the release of inflammatory mediators, including cytokines and chemokines [9,28,29,30], whose level is altered in the brain, CSF, and blood of AD patients [31,32,33].

Specifically, chemokines, whose primary role is regulation and enrollment of leukocytes to the site of inflammation, represent a very important class of inflammatory mediators involved in AD pathophysiology [34].

PROK2 has been demonstrated to be involved early in Aβ toxicity both in vitro, in rat cortical primary cultures and in vivo, in rat i.c.v. infused with Aβ, indicating that modulation of the prokineticin system could be a wide-ranging reaction to Aβ injury, at least in acute damage conditions [21,23].

Here, we demonstrated that PROK2 mRNA was significantly upregulated in both the prefrontal cortex and the hippocampus of Aβ_1-42_ injected rats, a widely used AD non-transgenic animal model, remaining at the same level up to 35 days after the infusion. This therefore indicates a long-term activity of Aβ toxic effect in PK system modulation. From immunofluorescence analysis, the increase in PROK2 immunoreactivity in response to Aβ insult has been established in both neurons and astrocytes, thus confirming that astrocytes are the main neural cells producing chemokines in response to Aβ [35,36,37].

Likewise, upregulation of PROK2 mRNA has been further observed in brain extracts from Tg2576 (TG) mice, a reliable transgenic model of AD, at both early and late stages, showing the greater significant PROK2 mRNA rise in in the cortex of pre-symptomatic stage mice. A rise in PROK2 content was also evident in hippocampal tissues from Tg2576 mice, although this was highly variable. Furthermore, in the hippocampus of adult Tg2576 mice, a deleterious role of PROK2 has been previously established in long-term potentiation (LTP) experiments, as revealed by the recovery of synaptic plasticity impairment obtained with the pharmacological blockade of prokineticin receptors [21]. These results indicate a detrimental role of this chemokine in memory processes, suggesting that targeting PROK2 receptors may offer innovative potential chances for treatment of AD, at least in animal models.

In view of the increased expression of PROK2 in AD transgenic mice, we extended our research to the human brain. This is the first observation describing the direct involvement of the PK system in Alzheimer’s disease, as demonstrated by the significant rise of PROK2 mRNA in the hippocampus of AD patients compared to age-matched, cognitively intact controls.

Interestingly, through a pilot study, we found a substantial increase of PROK2 levels in the serum of subjects affected by this pathology, which correlates with the rise here described in post-mortem brain tissues of AD patients.

These preliminary data suggest that such remote pathological events occurring at brain level could be also tracked in peripheral fluids of patients, opening the way for dedicated confirmatory studies.

In fact, numerous studies suggested neuroinflammation as a key pathway in AD, as plasma and brain levels of inflammatory proteins were reported to grow even before clinical onset of this pathology, generally with an inverse correlation in respect to AD progression [38,39].

An enhancement of MCP-1 (CCL2) concentration levels has been reported in CSF and serum of patients in the early stage of AD (MCI or mild AD) whereas, in the late stage, its amount is decreased in the serum but remains elevated in the CSF [40,41,42,43]. Likewise, CXCL8 (interleukin 8) has been found to be increased in both the serum and CSF of AD patients [40,44], as well as CXCL10 (IP-10), whose levels in CSF correlated with AD cognitive impairment [41].

A very recent multicentric study has indicated that numerous plasma proteins related to neuroinflammation, including three chemokines (eotaxin-1, MCP-1, and Macrophage Inflammatory Proteins (MIP) 1b (MIP-1b) and complement dysregulated proteins, could significantly and independently concur to differentiate MCI and AD patients from healthy controls. Results from this international study, involving a great number of subjects, indicated that both the chemokines eotaxin-1 and MCP-1 could be considered potential plasma biomarkers of the disease [16]

The results are in accordance with previous studies reporting that elevated MCP-1 and eotaxin-1 plasma levels are associated with a higher memory impairment in individuals with MCI and AD dementia, this damaging relationship being strongest when both inflammatory markers are raised [45].

However, results shown by Morgan and collaborators demonstrated that the absolute values of eotaxin-1 and MCP-1 plasma levels did not have significant differences, but could be considered AD pathological markers only by a logistic regression analysis which combines their values with age and apolipoprotein E (APOE) status.

Of note, unlike MCP-1 and eotaxin-1, whose plasma levels are only moderately altered in AD patients, results from our work showed an increase of about 1.6 times of PROK2 level in AD plasma in respect to controls. The results seem promising, although they require further validation in a more consistent number of patients.

Clearly, it will be very interesting to extend the study to MCI patients to conceivably identify a potential early disease biomarker, before the onset of significant cognitive change that may help, together with other altered parameters, to sub-classify the pathology in individual patients, allowing for a potential personalized treatment.

The upregulation of PROK2 in the brain of AD animal models and of patients and the crucial increase of its levels in the serum of AD patients could identify PROK2 as a potential blood-based biomarker of this pathology.

## Figures and Tables

**Table 1:** Gene primary sequence.

Species	Gene	Sequence
Rat	GAPDH	Fw: 5′-GCG AGA TCC CGC TAA CAT CAA ATG G-3′
		Rev: 5′-GCC ATC CAC AGT CTT CTG AGT GGC-3′
	
	PROK2	Fw: 5’-TCA TCA CCG GGG CTT GCG-3’
		Rev: 5’-TAA CTT TCC GAG TCA GGG-3’
Mouse	GAPDH	Fw: 5′-GCC AAG GCT GTG GGC AAG GT-3′
		Rev: 5′-TCT CCA GGC GGC ACG TCA GA-3′
	
	PROK2	Fw: 5′-CTC GGA AAG TTC CAT TTT GG-3′
		Rev: 5′-TTC CGG GCC AAG CAA ATA AAC C-3′
Human	β-actin	Fw: 5′-AGA TGA CCC AGA TCA TGT TTG-3′
		Rev: 5′-TAG ATG GGC ACA GTG TGG-3′
	
	PROK2	Fw: 5′-ATG TGC TGT GCT GTC AGT AT-3′
		Rev: 5′-AAA ATG GAA CTT TAC GAG TCA-3′
